# Diabetes Mellitus Accelerates Aβ Pathology in Brain Accompanied by Enhanced GAβ Generation in Nonhuman Primates

**DOI:** 10.1371/journal.pone.0117362

**Published:** 2015-02-12

**Authors:** Sachi Okabayashi, Nobuhiro Shimozawa, Yasuhiro Yasutomi, Katsuhiko Yanagisawa, Nobuyuki Kimura

**Affiliations:** 1 Tsukuba Primate Research Center, National Institute of Biomedical Innovation, 1–1 Hachimandai, Tsukuba-shi, Ibaraki, 305–0843, Japan; 2 The Corporation for Production and Research of Laboratory Primates, 1–1 Hachimandai, Tsukuba-shi, Ibaraki, 305–0843, Japan; 3 Section of Cell Biology and Pathology, Department of Alzheimer's Disease Research, Center for Development of Advanced Medicine for Dementia, National Center for Geriatrics and Gerontology (NCGG), Gengo 35, Moriika, Obu, Aichi, 474–8511, Japan; National Center of Neurology and Psychiatry, JAPAN

## Abstract

Growing evidence suggests that diabetes mellitus (DM) is one of the strongest risk factors for developing Alzheimer’s disease (AD). However, it remains unclear why DM accelerates AD pathology. In cynomolgus monkeys older than 25 years, senile plaques (SPs) are spontaneously and consistently observed in their brains, and neurofibrillary tangles are present at 32 years of age and older. In laboratory-housed monkeys, obesity is occasionally observed and frequently leads to development of type 2 DM. In the present study, we performed histopathological and biochemical analyses of brain tissue in cynomolgus monkeys with type 2 DM to clarify the relationship between DM and AD pathology. Here, we provide the evidence that DM accelerates Aβ pathology *in vivo* in nonhuman primates who had not undergone any genetic manipulation. In DM-affected monkey brains, SPs were observed in frontal and temporal lobe cortices, even in monkeys younger than 20 years. Biochemical analyses of brain revealed that the amount of GM1-ganglioside-bound Aβ (GAβ)—the endogenous seed for Aβ fibril formation in the brain—was clearly elevated in DM-affected monkeys. Furthermore, the level of Rab GTPases was also significantly increased in the brains of adult monkeys with DM, almost to the same levels as in aged monkeys. Intraneuronal accumulation of enlarged endosomes was also observed in DM-affected monkeys, suggesting that exacerbated endocytic disturbance may underlie the acceleration of Aβ pathology due to DM.

## Introduction

Alzheimer’s disease (AD) is a progressive neurological disorder that is histopathologically characterized by the formation of senile plaques (SPs) and neurofibrillary tangles (NFTs) [1, 2]. It is widely accepted that β-amyloid protein (Aβ), the major component of SPs, is a key molecule underlying AD pathogenesis [3, 4]. Several epidemiological/clinical studies have shown that diabetic mellitus (DM) patients are significantly more likely to develop cognitive dysfunction and exhibit increased susceptibility to AD [5–9], in consistent with the original Rotterdam study [10]. Recent findings also showed that there are several pathogenic connections between AD and DM patient brains, such as brain inflammation, mitochondrial dysfunction, and defective neuronal insulin signaling [11]. Insulin resistance causes alteration in GSK3β kinase signaling pathway as observed in AD brains, and it is also associated with an AD-like pattern of reduced cerebral glucose metabolic rate in brain [12, 13]. Moreover, accumulating evidences showed that the experimental induction of DM enhanced AD pathology even in rodents [14–25]. However, it remains unclear how DM physiologically accelerates AD pathology in the brain.

With advancing age, both SPs and NFTs occur spontaneously in brains of cynomolgus monkeys [26, 27]. In addition, the amino acid sequence of Aβ of cynomolgus monkeys is completely consistent with that of humans [28]. These advantages make this species a useful model to study age-dependent AD pathophysiology. As with humans, obesity occasionally occurs in adult, middle-aged monkeys, and it can result in the development of type 2 DM [29, 30]. Similar to the case of humans, these monkeys have a period of insulin resistance and hyperinsulinemia before developing overt DM, which is then accompanied by deficiency in pancreatic insulin production [29–31]. The pathological changes that occur in the pancreatic islets of aged monkeys are also similar to those seen in human diabetics, including the deposition of islet amyloid polypeptide (IAPP) [29–31]. In addition, gestational diabetes has been also reported in female cynomolgus monkeys [29–31]. Thus, cynomolgus monkeys are a useful species to investigate not only age-dependent AD lesions but also the relationship between DM and AD pathology.

Here, we report that DM enhances the generation of GM1-ganglioside-bound Aβ (GAβ) to accelerate SP deposition in cynomolgus monkey brains. GAβ was previously identified as the endogenous seed for Aβ fibril formation in the brain, and its generation is enhanced by endocytic disturbance, which is considered to be involved in early-stage AD pathology [32–34]. In DM-affected adult monkeys, the level of Rab GTPases in the brain was obviously increased as compared to normal adult monkeys, and intraneuronal endosomes were apparently enlarged. These findings suggest that DM exacerbates age-dependent endocytic disturbance, which then may lead to accelerate Aβ pathology via enhanced GAβ generation.

## Materials and Methods

### Animals

Forty-one cynomolgus monkey (*Macaca fascicularis*) brains were used in this study. Of these, six brains were from young monkeys (age: 6 and 7 years); six were from normal adult monkeys (age: 17, 18, 19, and 20 years); nine were from DM-affected adult monkeys (age: 17, 18, 19, and 20 years); ten were from normal aged monkeys (age: 24, 25, 26, and 28 years); and ten were from DM-affected aged monkeys (age: 24, 25, 26, and 28 years). The frontal and temporal lobes were used for immunohistochemical studies. The cerebral cortices of 12 monkeys were used for dot blot analyses. Of these 12, 3 were from young monkeys (age: 6 years [N = 2] and 7 years [N = 1]); 3 were from normal adult monkeys (age: 18 years, 19 years, and 20 years [N = 1 each]); 3 were from DM-affected adult monkeys (age: 18 years, 19 years, and 20 years [N = 1 each]); and 3 were from normal aged monkeys (age: 26 years [N = 2] and 28 years [N = 1]). The cerebral cortices of 12 female monkeys were used for Western blot analyses. Of these 12, 3 were from young monkeys (age: 7 years [N = 3]); 3 were from normal adult monkeys (age: 18 years, 19 years, and 20 years [N = 1 each]); 3 were from DM-affected adult monkeys (age: 18 years, 19 years, and 20 years [N = 1 each]); and 3 were from normal aged monkeys (age: 25 years and 26 years [N = 2]). The cerebral cortices of 14 female monkeys were used for Aβ ELISA. Of these 12, 4 were from young monkeys (age: 7 years [N = 4]); 3 were from normal adult monkeys (age: 18 years, 19 years, and 20 years [N = 1 each]); 3 were from DM-affected adult monkeys (age: 18 years, 19 years, and 20 years [N = 1 each]); and 4 were from normal aged monkeys (age: 24 years [N = 3], and 25 years [N = 1]). All brains were obtained from the Tsukuba Primate Research Center (TPRC), National Institute of Biomedical Innovation (NIBIO), Japan. Monkeys in the TPRC are reared in individual cages (0.5 m wide × 0.8 m high × 0.9 m deep; stain-less steel mesh). The breeding rooms are rectangular, and the individual cages are installed on the long sides of the room. Each room contains at least 90 cages. Therefore, monkeys can always make visual, auditory and olfactory contact with their roommates. Ambient temperature in the rooms is kept about 25°C, and humidity is set at 50% to 60%. The air is replaced 12 times hourly. Monkeys are provided with 100 g of apples in the morning, and 70 g of commercial food (Type AS; Oriental Yeast Co., Ltd., Tokyo, Japan) is given to them twice in the afternoon. Water is available ad libitum. Every morning their health status (e.g., viability, appetite, fur-coat appearance) was monitored by experienced animal technicians. When any abnormality is found, a veterinarian examines the monkey promptly and applies the appropriate treatment. Moreover, the monkeys are medically examined under anesthesia (ketamine hydrochloride) at least once every 2 y. The medical examination consists of body weight measurement, tuberculin test, blood sample, stool test, examination of the fundus, and a medicated bath. The maintenance of animals was conducted according to the rules for animal care of the TPRC at NIBIO for the care, use, and biohazard countermeasures of laboratory animals [35]. This study was carried out in strict accordance with the recommendations in the Animal Care and Use Committee of the NIBIO, Japan. The protocol was approved by the Committee on the Ethics of Animal Experiments of the NIBIO (DS17-001R1). When the monkey presents clinical symptoms by injury or illness and it could not expect recovery from pain or morbidity, it is judged as poor prognosis. In the present study, the animals used in this study died of natural causes were euthanized when they reached endpoints determined as poor prognosis. All DM-affected monkeys were also sacrificed because of poor prognosis. For euthanasia, the monkeys were deeply anesthetized with a lethal dose of pentobarbital, and all efforts were made to minimize suffering.

### Antibodies

In this study, we used the following antibodies: mouse monoclonal anti-dynein heavy chain (DHC) (Sigma, Saint Louis, MO); mouse monoclonal anti-dynein intermediate chain (DIC) (Millipore, Temecula, CA); mouse monoclonal anti- GAβ antibody (4396C) (22); mouse monoclonal kinesin light chain (KLC) (Santa Cruz Biotechnology, Santa Cruz, CA); mouse monoclonal anti-neprilysin (NEP; DAKO, Glostrup, Denmark); mouse monoclonal anti-phosphorylated tau antibody (AT8; Innogenetics, Gent, Belgium); mouse monoclonal anti-Rab5 antibody (Rab5m; Santa Cruz Biotechnology); rabbit polyclonal anti-Aβ antibody (IBL); rabbit polyclonal anti-full-length β-amyloid precursor protein (APP) antibody (IBL); rabbit polyclonal anti-Cathepsin D (CatD; Cell Signaling Technology, Danvers, MA); rabbit polyclonal anti-kinesin heavy chain (KHC) (Sigma); rabbit polyclonal anti-LC3 (Novus Biologicals, Littleton, CO); rabbit polyclonal anti-Rab5 antibody (Santa Cruz Biotechnology); rabbit polyclonal anti-Rab7 antibody (Sigma); rabbit polyclonal anti-Rab11 antibody (Santa Cruz Biotechnology); and rabbit polyclonal anti-synaptophysin (DAKO).

### Histopathological analyses for DM-associated pathology

Most of the tissues were fixed in 10% neutral buffered formalin, processed routinely and stained with hematoxylin and eosin (HE) for histopathological analyses. The pancreases were also stained with direct fast scarlet (DFS; Muto) for identification of islet amyloid.

### Immunohistochemistry

Brain samples were immersion-fixed in 10% neutral buffered formalin, embedded in paraffin, and cut into 4 μm-thick sections. For immunohistochemical analyses with anti-GAβ antibody, brain samples were fixed in 4% paraformaldehyde. Sections were deparaffinized by pretreatment with 0.5% periodic acid, and then incubated overnight at 4°C free floating in the following primary antibody solutions: (1) Aβ (1:100); (2) APP (1:100); (3) Rab5 (1:100); (4) GAβ (1:20); or (5) AT8 (1:100). Following brief washes with buffer, the sections were sequentially incubated with biotinylated goat anti-mouse IgG (1:200) or goat anti-rabbit IgG (1:200), followed by streptavidin-biotin-horseradish peroxidase complex (DAKO). Immunoreactive elements were visualized by treating the sections with 3–3' diaminobenzidine tetroxide (Dojin Kagaku). The sections were then counterstained with hematoxylin. The immunoreactivity of Aβ, GAβ, APP or Rab5 in a given cortical area was quantified with computer software Image J 1.49i (National Institue of Health). For quantification analyses, the sections were not counterstained with hematoxylin.

### Biochemical analyses of monkey brains

Frozen monkey brain tissue (wet weight 0.2 g) was homogenized in a glass homogenizer with 4 ml of homogenate buffer solution (0.32 M sucrose, 10 mM Tris-HCl [pH 7.6], 0.25 mM PMSF, and 1 mM EDTA, and Complete Mini proteinase inhibitor cocktail), and then centrifuged at 100,000xg for 20 min to obtain the supernatant fraction. The proteins in the fraction were subjected to dot blot analyses. Brain homogenates were also centrifuged at 1,000×g for 10 min to remove the nuclear fraction, and then the supernatant was centrifuged at 105,000×g for 60 min to obtain the microsomal fraction. The proteins in the microsomal fraction were subjected to Western blot analyses. For Aβ ELISA, brain homogenates were incubated with Triton-X100 at a final concentration of 1% for 15 min at 37°C, and spun at 100,000 × g for 20 min at 20°C. The pellet was homogenized in homogenate buffer containing 1% sarkosyl, incubated for 15 min at 37°C, and spun at 100,000 × g for 20 min at 20°C. The sarkosyl-insoluble pellet was sonicated in 70% folic acid, cleared by centrifugation at 100,000 × g for 20 min at 20°C. The supernatant was evaporated, and then resuspended in dimethyl sulfoxiside (Sigma).

### Dot blot analyses

Dot blot analyses were performed to assess age- and DM-related changes in GAβ formation. Brain samples were adjusted to 1 mg, 2.5 mg, and 5 mg, and then applied onto nitrocellulose membranes and dried. The membranes were blocked with 5% nonfat dried milk in 20 mM PBS (pH 7.0) and 0.1% Tween-20 for 1 h at room temperature, and then incubated in the anti-GAβ antibody solution overnight at 4°C. They were then incubated with horseradish peroxidase-conjugated goat anti-mouse IgG (1:10000; Cell Signaling Technology) for 1 h at room temperature. Immunoreactive elements were visualized using enhanced chemiluminescence (Luminata Forte Western HRP Substrate, Millipore).

### Western blot analyses

Western blot analyses were performed to assess age- and DM-related changes in APP, Rab5, Rab7, Rab11, DHC, DIC, KHC, KLC, NEP, Cathepsin D heavy chain (CatD HC), and LC3 expression. Each microsome fraction prepared as described above was adjusted to 5 mg, and then analyzed using SDS-polyacrylamide gel electrophoresis using 14% (for LC3) or 12.5% (other proteins) acrylamide gels. Separated proteins were blotted onto polyvinylidense fluoride membranes (Immobilon P; Millipore). The membranes were blocked with 5% nonfat dried milk in 20 mM PBS (pH 7.0) and 0.1% Tween-20 for 1 h at room temperature, and then incubated overnight at 4°C in the following primary antibody solutions: (1) synaptophysin (1:20000); (2) APP (1:2000); (3) Rab5m (1:2000); (4) Rab7 (1:10000); (5) Rab11 (1:2000); (6) DHC (1:1000); (7) DIC (1:20,000); (8) KHC (1:5,000); (9) KLC (1:1000); (10) NEP (1:4,000); (11) CatD (1:2,000); or (12) LC3 (1:2,000). They were then incubated with either horseradish peroxidase-conjugated goat anti-mouse IgG or goat anti-rabbit IgG (1:10,000; Cell Signaling Technology) for 1 h at room temperature. Immunoreactive elements were visualized using enhanced chemiluminescence. To confirm reproducibility, immunoreactive bands obtained from the Western blots were quantified using commercially available software (Quantity One; PDI, Inc.).

### Aβ ELISA

Total Aβ levels in young monkey, normal adult monkey, DM-affected monkey, and normal aged monkey brains were determined using a sandwich ELISA. The kit for Aβ (1-X) was obtained from IBL (Gunma, Japan). The ELISA assay was carried out according to the instruction manual. All samples were measured in duplicate.

### Data analyses

Data obtained from quantitative analyses of immunohistochemistry and biochemical analyses are shown as means ± SD. For statistical analyses, one-way ANOVAs were performed, followed by the Fisher’s post hoc test.

## Results

### Clinical background and DM-associated pathology in cynomolgus monkeys

Tsukuba Primate Research Center (TPRC) maintains a large breeding and rearing colony of cynomolgus monkeys for high-quality production of nonhuman primate models and biomedical investigations. In the TPRC colony, some adult monkeys are spontaneously affected with type 2 DM for various reasons, such as pregnancy history and environmental factors. The clinical background of all monkeys used for this study is shown in (S1 Table). TPRC has accumulated clinical data for more than 40 years. Based on these data, the normal blood glucose level for female monkeys is in the range of 24 to 74 mg/dL, and for male monkeys the range is 24 to 76 mg/dL. Normal blood triglyceride levels are in the range of 8 to 85 mg/dL for females, and 6 to 52 mg/dL for males. The blood glucose and triglyceride levels of normal adult monkeys used in the present study were all in the normal range, and their body weights were in the range of historical TPRC data for normal monkeys (S1 Table). All DM-affected monkeys, on the other hand, exhibited hyperlipidemia and hyperglycemia (S1 Table). These DM-affected monkeys were severely obese; however, their body weight at acquisition of their brains for this study had decreased to about less than half the maximum body weights they attained during their lifetime (S1 Table).

**Fig 1:**
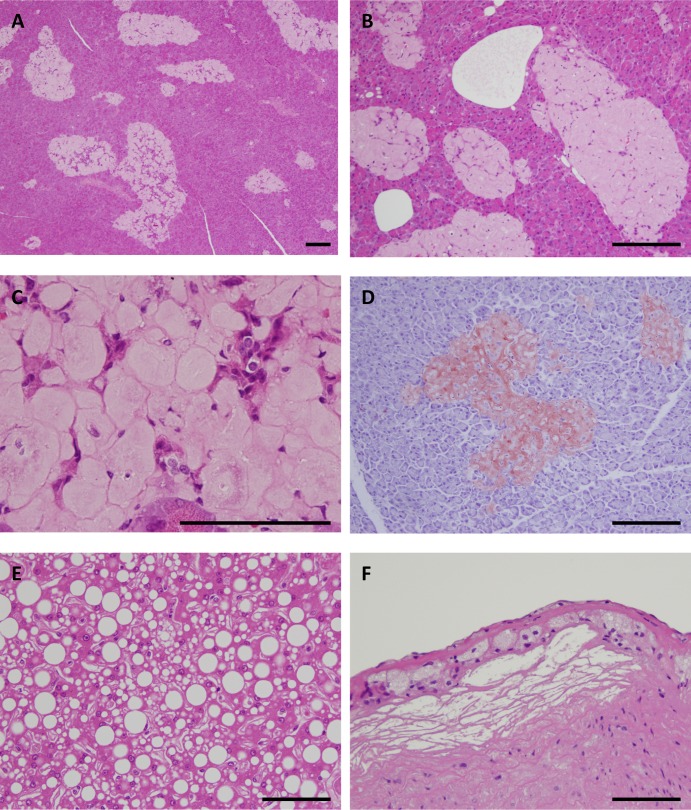
Histopathology of adult monkeys with type 2 diabetes mellitus. (A) Hematoxylin-eosin (HE)-stained section of the pancreas from a 17-year-old cynomolgus monkey with type 2 diabetes mellitus (DM) (S1 Table, Number 1). Most of the islets were replaced by abundant amyloid deposits. (B) HE-stained section of the pancreas from an 18-year-old cynomolgus monkey with DM (S1 Table, Number 3). Most of the islets were replaced with severe amyloid deposits. (C) HE-stained section of the pancreas from a 19-year-old cynomolgus monkey with DM (S1 Table, Number 7) showing hyalinized islets. Very few islet cells remain. (D) Direct fast scarlet-stained section of pancreas from an 18-year-old cynomolgus monkey with DM (S1 Table, Number 5). Hyalinized islets with severe amyloid deposition were positive for direct fast scarlet staining. (E) HE-stained section of the liver from an 18-year-old cynomolgus monkey with DM (S1 Table, Number 3). Marked fatty degeneration was observed in the liver. (F) HE-stained section of the aorta from an 18-year-old cynomolgus monkey with DM (S1 Table, Number 5). Mild atheromatosis with foam cells and sterol clefts was observed in the aorta. Scale bars for a-f, 100 μm.

Histopathologically, islet amyloid was found in the pancreases of all DM-affected adult monkeys (Fig. 1A). In eight DM cases, most hyalinized islets were replaced with severe amyloid deposits, with the remaining islet cells being severely degenerated or decreased in apparent number (Fig. 1A-C). The hyalinized islets with severe amyloid deposits stained positive for direct fast scarlet (DFS) (Fig. 1D). Furthermore, two DM cases had fatty degeneration of the liver, and one DM case had mild atheromatosis. Thus, histopathological analyses demonstrated that these adult monkeys clearly had DM. On the other hand, we did not observe apparent vascular lesions in all DM monkey brains.

### DM accelerates Aβ pathology in cynomolgus monkey brain

**Fig 2:**
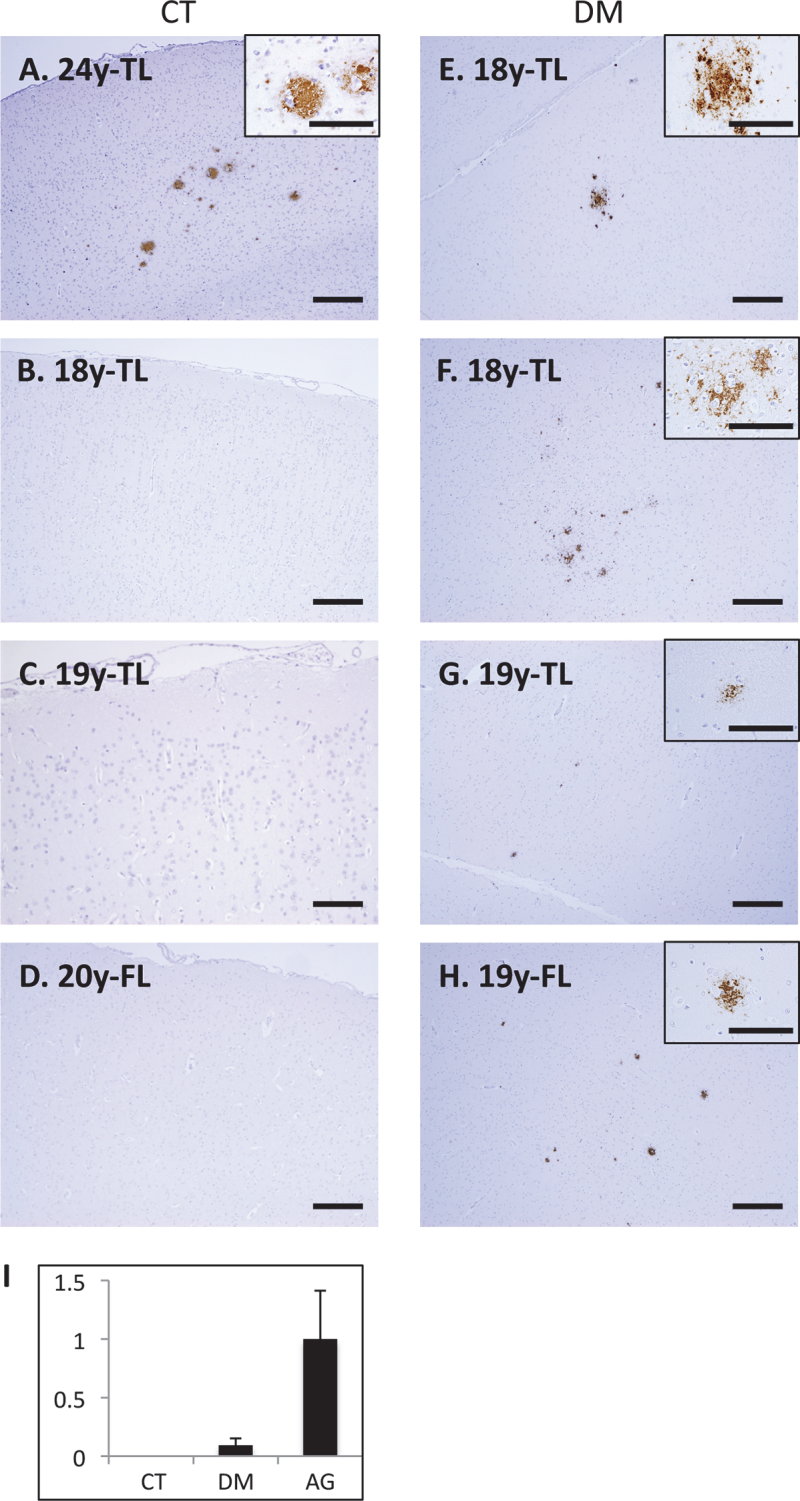
Senile plaques in the brains of adult monkeys with DM. Images of temporal lobe (TL) and frontal lobe (FL) sections from normal cynomolgus monkeys (A-D) and cynomolgus monkeys with DM (E-H). Sections were immunostained with anti-Aβ antibody and counterstained with hematoxylin. In aged monkey brains, we observed SPs immunostained with anti-Aβ antibody, as previously reported (A). In contrast, we did not observe Ab-immunopositive structures in the normal adult monkey brains (B-D). However, we did observe small but obvious Aβ-immunopositive senile plaques (SPs) in the frontal and temporal cortices of DM-affected adult monkeys (E-H). Scale bars, 100 μm. (I) Quantitative image analysis of Ab-immunopositive area in the sections obtained from female normal adult monkey, DM-affected adult monkey, and normal aged monkey brains. Data obtained from normal aged monkey brains were set as standards. Y-axes show the mean values of the quantified data. CT, normal cynomolgus monkeys. DM, DM-affected monkeys.

**Fig 3:**
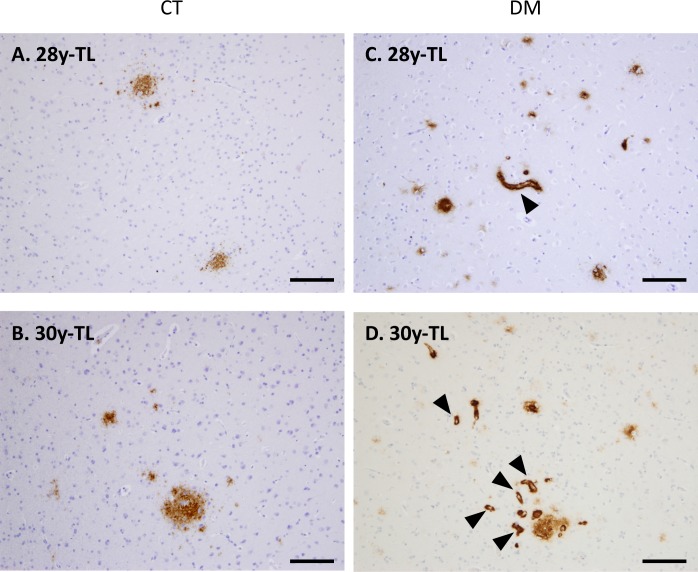
Cerebral amyloid angiopathy in the brains of aged monkeys with DM. Images of temporal lobe (TL) sections from normal cynomolgus monkeys (A, B) and cynomolgus monkeys with DM (C, D). Sections were immunostained with anti-Aβ antibody and counterstained with hematoxylin. In the brains of DM-affected aged monkeys, we observed very severe CAA lesions (arrowheads) as compared to normal aged monkeys. CT, normal aged monkeys. DM, DM-affected aged monkeys. Scale bars, 100 μm.

Next, we conducted immunohistochemical analyses to assess whether DM affects AD pathology in these cynomolgus monkey brains. As previously reported [26, 36], we observed SP depositions in the brains of aged monkeys (Fig. 2A), but not in those of normal adult monkeys younger than 20 years of age (Fig. 2B-D). Strikingly, we apparently observed diffuse Aβ-immunopositive SPs in brains of six DM-affected adult monkeys younger than 20 years old, even though they were very small quantities as compared to aged monkey brains (Fig. 2E-I). In aged cynomolgus monkey brains, cerebral amyloid angiopathy (CAA) lesions are also observed, and NFTs are observed over 32-year-old monkey brains [26, 27]. Although abnormally phosphorylated tau accumulation was not observed, we found much severe CAA in the brains of aged monkeys with DM (Fig. 3).

### DM enhances GAβ generation in adult monkey brains

**Fig 4:**
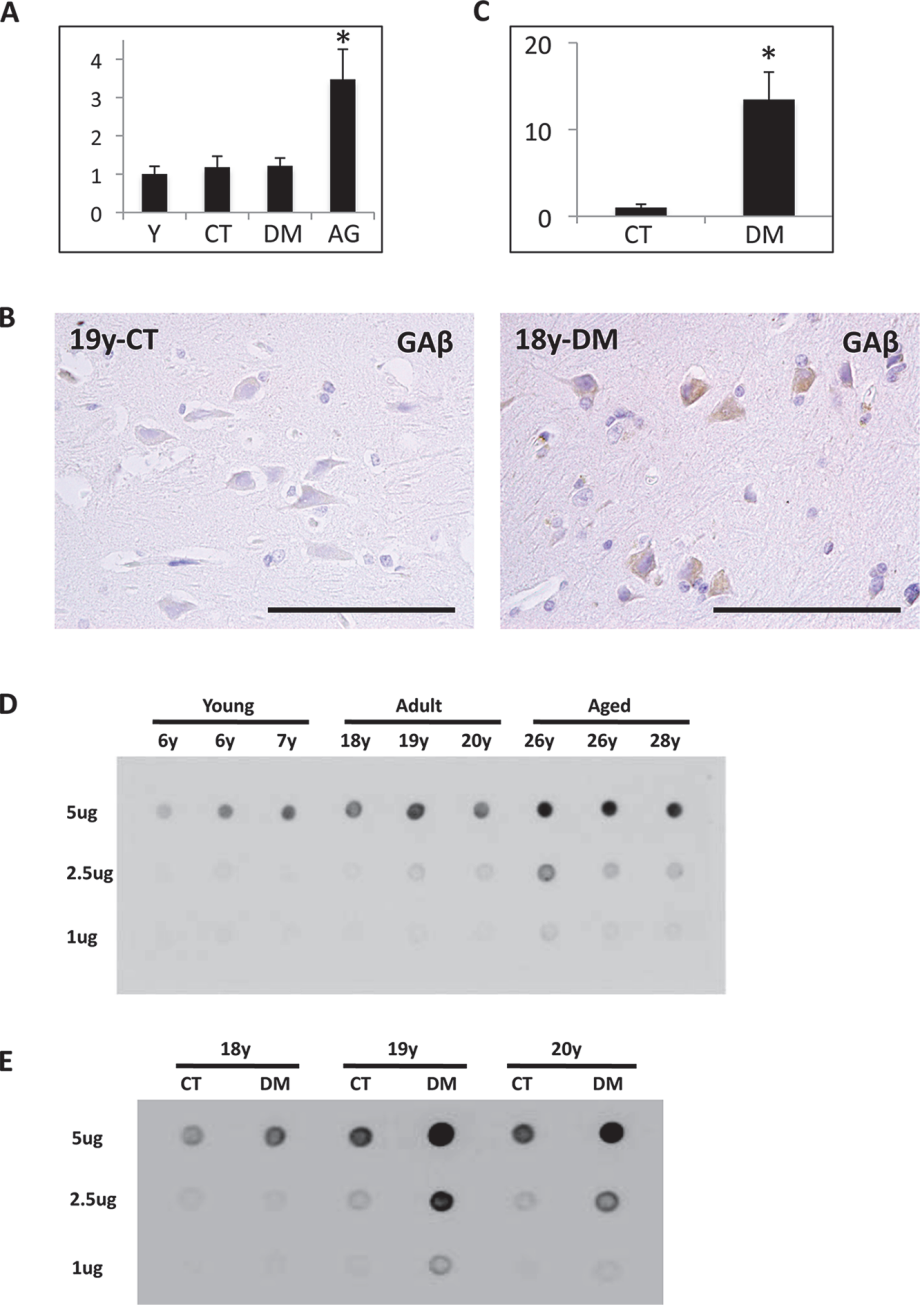
The analyses of Aβ and GAβ in the brains of normal and DM-affected monkeys. (A) Aβ level in young monkey, normal adult monkey, DM-affected monkey, and normal aged monkey brains were assessed with sandwich ELISA. Aβ level was significantly increased in normal aged monkey brains. In DM-affected monkey brains, Aβ level seemed unchanged. Data obtained from young monkey brains were set as standards. Y-axes show the mean values of the quantified data. Values are means ± SD. *P < 0.02. (B) Image of temporal lobe sections from a 19-year-old normal adult monkey and an 18-year-old cynomolgus monkey with DM. Sections were immunostained with the anti-GAβ-specific antibody 4396C and counterstained with hematoxylin. In the brain of the normal adult monkey, we observed little, if any, immunoreactivity for anti-GAβ antibody. By contrast, in the brain of the DM-affected adult monkey, we observed several neurons immunopositive for anti-GAβ antibody. Scale bars, 100 μm. (C) Quantitative image analysis of GAb-immunopositive area in the sections obtained from female normal adult monkey and DM-affected adult monkey brains. Data obtained from normal adult monkey brains were set as standards. Y-axes show the mean values of the quantified data. Values are means ± SD. *P < 0.02. (D) Dot blots showing the amount of GAβ generated in brains of cynomolgus monkeys of different ages. The blot samples were adjusted to 1 μg, 2.5 μg, or 5 μg of total protein. Dot blot analyses showed that GAβ generation increased in an age-dependent manner. (E) Dot blots showing the amount of GAβ generated in the brains of normal adult monkeys and DM-affected adult monkeys. The blot samples were adjusted to 1 μg, 2.5 μg, or 5 μg of total protein. The amount of GAβ in brains samples from DM-affected monkeys was significantly increased compared to those from normal adult monkeys. CT, normal adult monkeys; DM, DM-affected adult monkeys.

To assess whether DM affects Aβ level in monkey brains, we examined Aβ ELISA analyses. In aged monkey brains, Aβ level was significantly increased, suggesting that the increase of Aβ level correlates with age-dependent SP depositions (Fig. 4A). However, surprisingly, Aβ level was just slightly increased in DM-affected adult monkey brains as compared to normal adult monkey brains (Fig. 4A). We previously identified a unique Aβ species, called GAβ, characterized by its binding to GM1 ganglioside; GAβ was demonstrated in brain tissue along with early pathological changes characteristic of AD [32]. Accumulating evidence suggests that GAβ accelerates Aβ fibril formation by acting as a seed molecule [37–40]. We have also confirmed age-dependent GAβ generation in cynomolgus monkey brains [32, 41].

To test our hypothesis that DM enhances GAβ generation in the brain, we performed additional immunohistochemical analyses using an anti-GAβ-specific antibody. In the brains of normal adult monkeys, we observed little, if any, immunoreactivity with anti-GAβ antibody (Fig. 4B). In contrast, in the brains of DM-affected adult monkeys, we frequently observed neurons clearly immunopositive for anti-GAβ antibody (Fig. 4B). Quantitative analyses also confirmed that the immunoreactivity of GAβ was significantly increased in DM-affected adult monkey brains (Fig. 4C).

For biochemical analyses, we carried out dot blot analyses on monkey brain samples. Because of its conformational features, GAβ is not recognized by western blot analyses. Our dot blot analyses showed that GAβ generation increased in an age-dependent manner in cynomolgus monkey brains (Fig. 4D). GAβ immunoreactivity was much stronger in aged monkey brains, indicating that the amount of GAβ generated generally parallels age-dependent SP deposition as well as Aβ level. In normal adult monkey brains, the amount of GAβ generation was not much different from that in young monkey brains (Fig. 4D). It is noteworthy that GAβ generation was significantly increased in DM-affected adult monkey brains (Fig. 4E).

### DM exacerbates endocytic disturbance with significant increase of Rab GTPases

In the brains of early-stage AD patients, neuronal endocytic pathology, such as intracellular accumulation of abnormally enlarged endosomes, is frequently observed [42–44]. We previously demonstrated that aging causes endocytic pathology along with a significant increase in Rab GTPases, resulting in the intracellular accumulation of APP [45]. This endocytic disturbance was observed in brains from cynomolgus monkeys almost 10 years before SP deposition begins [46]. We also reported that GAβ accumulates in enlarged endosomes of neurons in aged cynomolgus monkey brains [33]. Moreover, endocytic disturbance induces GAβ generation [34]. Taken together, these findings suggest that endocytic disturbance is involved in age-dependent Aβ pathology. Thus, to assess whether DM enhances endocytic disturbance, we investigated endocytic pathology in monkey brains.

**Fig 5:**
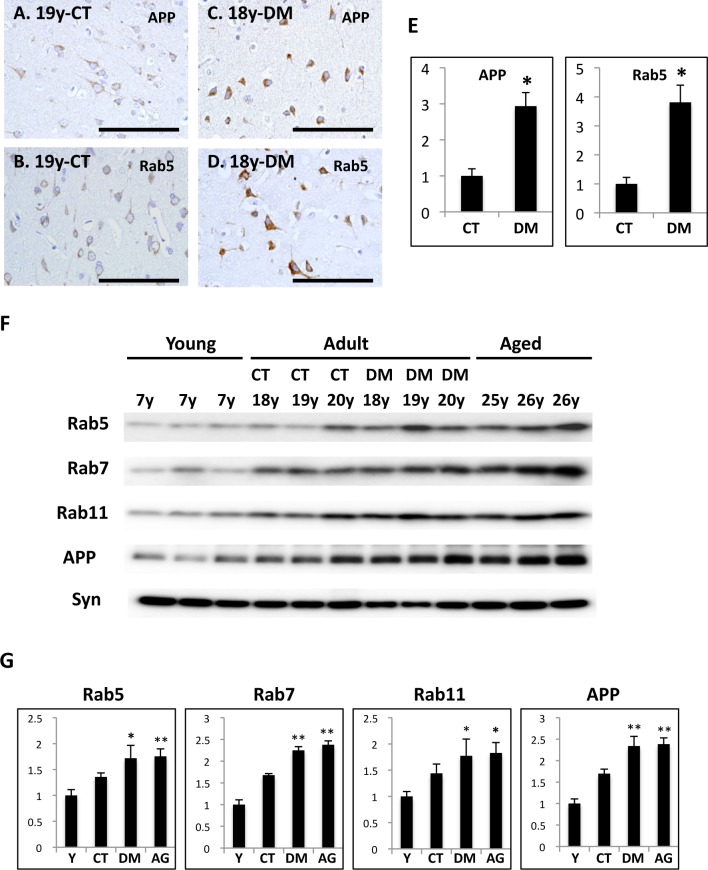
Immunohistochemistry and Western blot analyses of Rab5, Rab7, Rab11, and APP in the brains of normal and DM-affected monkeys. Images of temporal lobe sections from a 19-year-old normal cynomolgus monkey (A, B) and an 18-year-old cynomolgus monkey with DM (C, D). Sections were immunostained with anti-APP antibody (A, C) or anti-Rab5 antibody (B, D), and then counterstained with hematoxylin. Scale bars, 100 μm. In the brains of normal adult monkeys, APP and Rab5 were observed as small granules in neurons (A, B). By contrast in the brains of DM-affected adult monkeys, APP- and Rab5-immunopositive granules were enlarged, and APP and Rab5 immunoreactivity was significantly more robust (C, D). (E) Quantitative image analysis of APP or Rab5-immunopositive area in the sections obtained from female normal adult monkey and DM-affected adult monkey brains. Data obtained from normal adult monkey brains were set as standards respectively. Y-axes show the mean values of the quantified data. Values are means ± SD. *P < 0.01. (F) Western blots showing the amounts of APP, Rab5, Rab7, Rab11, and synaptophysin in the brains of normal monkeys and DM-affected monkeys of different ages. Western blot analyses showed that APP and Rab GTPases were significantly increased in both DM-affected adult and aged monkey brains. In the brains of DM-affected adult monkeys, APP and Rab GTPases levels were obviously increased compared to those of normal adult monkeys. Lanes contained microsome fractions derived from the brains of young monkeys, normal adult monkeys, DM-affected adult monkeys, and aged monkeys. CT, normal adult monkeys; DM, DM-affected adult monkeys. (G) Age-related and DM-related changes in APP, Rab5, Rab7, and Rab11 in cynomolgus monkey brains. Data obtained from young monkey brains were set as standards; *P<0.05 **P<0.01. Y-axes show the mean values of the quantified data. Y, young monkeys; CT, normal adult monkeys; DM, DM-affected adult monkeys; AG, normal aged monkeys.

In the brains of normal adult monkeys, APP and Rab5-positive early endosomes were observed as small granules in neurons (Fig. 5A, B). By contrast, in the brains of DM-affected adult monkeys, the immunoreactivity of APP and Rab5 was significantly stronger and the immunopositive granules were larger (Fig. 5C, D). Quantitative analyses confirmed that both APP- and Rab5-immunopositive granules were significantly increased in DM-affected adult monkey brains as compared to normal adult monkey brains (Fig. 5E). Western blot analyses showed that Rab5, Rab7 (late endosome-associated GTPase), and Rab11 (recycling endosome-associated GTPase) were increased in aged monkey brains, corroborating that age-dependent endocytic disturbance does occur in monkey brains, as previously reported (Fig. 5F, G) [45]. The amount of APP was also significantly increased in aged monkey brains as previously reported (Fig. 5F, G) [45, 47]. In the brains of DM-affected adult monkeys, Rab GTPases and APP levels were apparently increased compared to the brains of normal adult monkeys, being almost same as in aged monkey brains (Fig. 5F, G).

### DM affects cathepsin D level and autophagosome clearance

**Fig 6:**
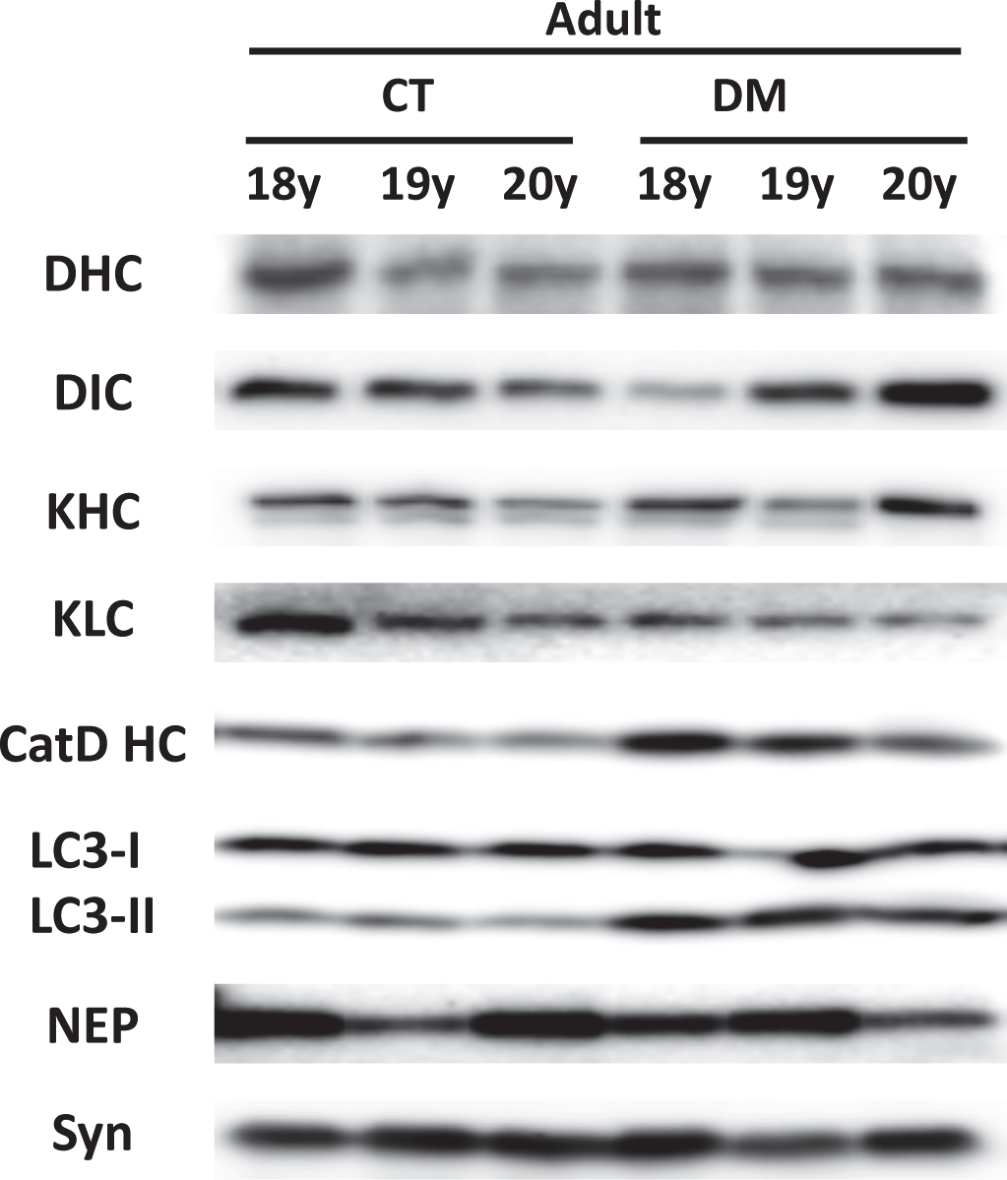
Western blot analyses of axonal motor proteins, cathepsin D heavy chain, autophagosome marker LC3, and neprilysin in the brains of normal and DM-affected adult monkeys. Western blots showing the amounts of axonal motor proteins, cathepsin D heavy chain (CatD HC), autophagosome marker LC3, and neprilysin (NEP) in the brains of normal and DM-affected adult monkeys. Western blot analyses showed that the level of axonal motor proteins such as dynein heavy chain (DHC), dynein intermediate chain (DIC), kinesin heavy chain (KHC), and kinesin light chain (KLC) unchanged. The level of CatD HC increased in DM-affected monkey brains, and LC3-II showed significant increase in DM-affected adult monkeys. We did not observed DM-related changes in LC3-I and neprilysin (NEP) level. CT, normal adult monkeys; DM, DM-affected adult monkeys.

Endosome trafficking is mediated by axonal transport motor proteins [48], and a recent study showed that the experimental induction of DM alters axonal motor protein levels in rodent model [49]. In the present study, we did not find apparent differences in axonal motor protein levels between normal and DM-affected adult monkey brains (Fig. 6). Endocytic disturbance is also induced by the breakdown in lysosomal degradation [50]. In DM-affected adult monkey brains, the level of cathepsin D (CatD) heavy chain increased (Fig. 6). On the other hand, we observed the significant increase in autophagosome marker LC3-II level without any alterations in LC3-I level (Fig. 6). A recent study also showed that DM-associated down-regulation of insulin signals reduces the level of Aβ degrading enzymes such as neprilysin (NEP) [51]. However, we did not find a clear reduction tendency in NEP levels in the brains of DM-affected adult monkeys (Fig. 6).

## Discussion

Here, we present the evidence that DM accelerates Aβ pathology in the brain parenchyma of nonhuman primates, which have not undergone any genetic manipulation. We demonstrated that DM does so by enhancing the generation of GAβ, the endogenous seed for Aβ fibril formation in the brain. The brains of DM-affected adult monkeys contained robust endocytic pathology, such as a significant increase in Rab GTPases and intraneuronal accumulation of enlarged endosomes. Endocytic disturbance is a cellular pathological characteristic of neurons of AD patients and enhances GAβ generation [33, 34]. Thus, our present findings suggest that DM exacerbates age-dependent endocytic disturbance, which in turn enhance GAβ generation resulting in accelerated Aβ pathology.

Recent epidemiological/clinical studies suggest that DM is a major risk factor for developing AD [5–9]. However, the underlying mechanisms for this association remain unclear. Thus, in the present study, we performed histopathological and biochemical analyses using brains from DM-affected cynomolgus monkeys in order to assess the relationship between DM and AD pathology. As previously reported, SPs spontaneously form in the brains of aged monkeys over the age of 25 years, but never in the brains of normal young monkeys and adult monkey younger than 20 years (Fig. 2A-D) [26]. Strikingly, our immunohistochemical analyses revealed SP depositions in the brains of DM-affected adult monkeys as young as 18 years (Fig. 2E-H). To our knowledge, this is the first study to show that DM enhances Aβ pathology even in nonhuman primate brains without genetic manipulation. We also observed much severe CAA lesions in the brains of DM-affected aged monkeys than in those of normal aged monkeys (Fig. 3). These findings are consistent with the previous studies showing that DM-related conditions induce amyloidogenesis and Aβ pathology in rodent models [14–25]. Although additional studies are needed, these findings suggest that DM can induce not only parenchymal Aβ pathology but also vascular Aβ pathology in an age-dependent manner.

To clarify the mechanism of how DM enhances Aβ pathology in the brain, we also assessed the amount of Aβ and GAβ, a seed molecule for Aβ aggregation [32]. Intriguingly, Aβ level was not so much increased in DM-affected adult monkey brains, in contrast to aged monkey brains (Fig. 4A). In DM-affected adult monkey brains, SP depositions were quite small quantities (Fig. 2I), and a couple of more years can induce age-dependent SP depositions in normal adult monkey brains [26, 36]. That may be why we could not find the significant increase of Aβ level between DM-affected adult monkey and normal adult monkey brains. On the other hand, both immunohistochemical and dot blot analyses demonstrated that the amount of GAβ was clearly increased in the brains of DM-affected adult monkeys compared to control adult monkey brains (Fig. 4B-E). These findings strongly suggest that the acceleration of GAβ generation might be responsible for the early deposition of SPs in the brains of DM-affected adult monkeys. Moreover, the result of this study also suggests that enhanced Aβ aggregation could induce SP deposition without significant changes in total Aβ level. Relevant to proposed AD pathophysiogical mechanisms, we also observed apparent endocytic pathology, including enlarged early endosomes and APP accumulation in neurons of DM-affected adult monkeys (Fig. 5A-E). Western blot analyses confirmed a significant increase of Rab GTPases in these brains at nearly the same level as in aged monkey brains (Fig. 5F, G). Our previous studies showed that an increase in Rab GTPases is a good indicator for alterations in intracellular endosome trafficking associated with a particular Rab GTPase [45, 46]. Indeed, increased Rab GTPase levels are strongly associated with endocytic disturbance [45, 46]. The observation that experimentally induced disorders of the endocytic pathway cause GAβ-dependent Aβ pathology [34, 52] supports the premise that endocytic disturbance is likely responsible for enhanced GAβ generation. Along these lines, we surmise that intracellular endosome trafficking would be altered in the brains of DM-affected adult monkeys, resulting in severe endocytic disturbance, as observed in aged monkey brains. This might be why GAβ generation was enhanced, thereby inducing SP deposition (Fig. 2). Moreover, the results of this study strongly support the idea that endocytic disturbance is essentially involved in the development of AD pathology [33, 34, 42–45].

A recent study showed that the expression of axonal transport motor proteins was altered in experimentally DM-induced rodent model, and axonal transport motor proteins are indeed required for endosome trafficking [48, 49]. However, in the present study, we did not find any changes in axonal motor protein levels, suggesting that the mechanism underlying endocytic disturbance in the brains of DM-affected adult monkeys would be independent of axonal motor protein levels. Previous finding showed that the breakdown in lysosomal degradation also induces endocytic disturbance [50]. In DM-affected adult monkey brains, the level of CatD heavy chain increased in DM-affected adult monkey brains, indicating that the endosomal-lysosomal system is activated as such in AD patient brains (Fig. 6) [53]. This finding suggests that DM really enhances AD pathology. On the other hand, we observed the significant increase in autophagosome marker LC3-II level in DM-affected adult monkey brains (Fig. 6). Since LC3-I level was unchanged, the induction of autophagy was not altered, but lysosomal-autophagosome clearance was likely disturbed in DM-affected adult monkey brains (Fig. 6). The defective lysosomal-autophagosome clearance is associated with AD pathology [50, 54–56], and the result of this study is also consistent with a previous finding that the aberrant lysosomal-autophagic turnover is associated with the accumulation of GAβ in rodent brain [57].

Given that CatD heavy chain level was increased, i.e. lysosomal degradation was induced (Fig. 6), the disturbance in the fusion of autophagosome and lysosome might be responsible for impaired lysosomal-autophagosome clearance in DM-affected adult monkey brains. The fusion step is indispensable for lysosomal-autophagosome clearance [58, 59] and mediated by Rab7 [60]. In DM-affected adult monkey brains, Rab7 level was obviously increased as compared to normal adult monkey brains, indicating that Rab7-mediated transport was really disturbed. Growing evidences suggest that membrane-bound phosphoinositides regulate Rab-mediated endosome trafficking [61, 62], and the metabolism of phosphoinositides was affected by the disruption of insulin signaling [63–65]. Recent studies also showed that Rab activity is affected by insulin signaling and that PI3K inhibition causes upregulation of Rab5 [66, 67]. In the present study, we observed amyloid deposition in the pancreatic islets of all adult monkeys with DM. The remaining islet cells were severely degenerated and few in number, all characteristics of DM pathology in humans. These pancreatic pathologies suggest that insulin signaling also would be greatly impaired in the brains of DM-affected adult monkeys (Fig. 1A-D). Thus, although additional investigations are needed, impaired insulin signaling would exacerbate age-related endocytic disturbances via such alteration in the metabolism of phosphoinositides and/or Rab GTPases, inducing GAβ generation and ultimately resulting in enhanced Aβ pathology. It is reasonable idea because of the fact that insulin resistance is the core defect in DM [68]. In the brains of DM-affected adult monkeys, NEP levels were not affected (Fig. 6), suggesting that the enhanced SP deposition we observed is not due to disturbances in Aβ degradation by NEP.

**Fig 7:**
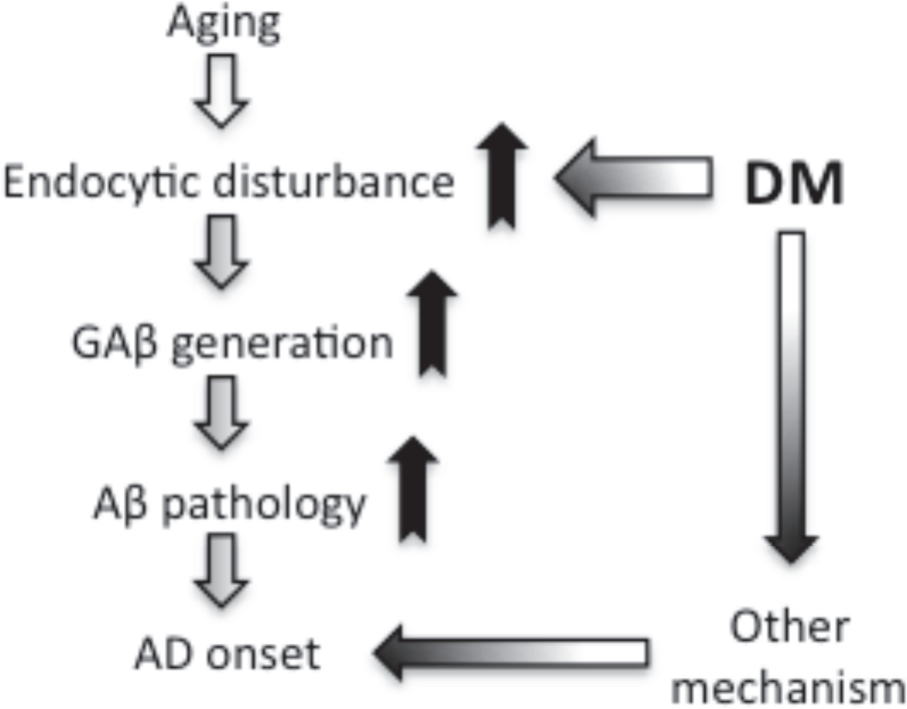
Hypothetical schema of DM-induced Aβ pathology leading to AD onset. From the results of this study, we propose that DM induces GAβ generation by exacerbating age-dependent endocytic disturbance, resulting in enhanced Aβ pathology in the brain. Although additional studies are needed to clarify the whole mechanisms underlying DM-associated pathology, we hypothesize that, at the very least, enhanced Aβ pathology accompanied by endocytic disturbance might be involved in the development of AD.

In conclusion, we provide evidence that DM induces GAβ generation and accelerates Aβ pathology *in vivo* in cynomolgus monkey brains. Since the amino acid sequence of cynomolgus monkey Aβ corresponds completely with that of human Aβ, it is reasonable that the enhanced Aβ pathology we observed in monkeys with DM should also occur in humans with DM. Moreover, our present study showed that DM could also exacerbate endocytic disturbance. Although additional studies are needed to determine more precisely the mechanisms responsible for enhanced Aβ pathology in the brains of DM-affected monkeys, our findings suggest that DM may exacerbate age-dependent endocytic disturbance, leading to enhanced GAβ generation and Aβ fibril formation (Fig. 7). Importantly, several studies showed that Aβ impairs insulin signaling itself [69–71], and then it may lead to aggravate the insulin resistance-related AD pathology [11–13]. Thus enhanced Aβ pathology would contribute to DM-induced AD pathogenesis with such other mechanism (Fig. 7). Moreover, DM may also alter neuronal activity by exacerbating endocytic disturbance as we previously reported [46]. Hence, a reasonable therapeutic strategy to prevent the development of AD pathology is to treat or prevent DM.

## Supporting Information

S1 ARRIVE Checklist The ARRIVE Guidelines Checklist. (PDF)

S1 Table Clinical Background of monkeys analyzed in the present study. (XLSX)
